# Path Curvature Discrimination: Dependence on Gaze Direction and Optical Flow Speed

**DOI:** 10.1371/journal.pone.0031479

**Published:** 2012-02-29

**Authors:** Colas N. Authié, Daniel R. Mestre

**Affiliations:** 1 Aix-Marseille Univ, UMR 7287 Institut des Sciences du Mouvement, Marseille, France; 2 CNRS, UMR 7287 Institut des Sciences du Mouvement, Marseille, France; University of Alberta, Canada

## Abstract

Many experimental approaches to the control of steering rely on the tangent point (TP) as major source of information. The TP is a good candidate to control self-motion. It corresponds to a singular and salient point in the subject's visual field, and its location depends on the road geometry, the direction of self-motion relative to the road and the position of the driver on the road. However, the particular status of the TP in the optical flow, as a local minimum of flow speed, has often been left aside. We therefore assume that the TP is actually an optimal location in the dynamic optical array to perceive a change in the trajectory curvature. In this study, we evaluated the ability of human observers to detect variations in their path curvature from optical flow patterns, as a function of their gaze direction in a virtual environment. We simulated curvilinear self-motion parallel to a ground plane. Using random-dot optic flow stimuli of brief duration and a two-alternative forced-choice adaptive procedure, we determined path curvature discrimination thresholds, as a function of gaze direction. The discrimination thresholds are minimal for a gaze directed toward a local minimum of optical flow speed. A model based on Weber fraction of the foveal velocities (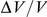) correctly predicts the relationship between experimental thresholds and local flow velocities. This model was also tested for an optical flow computation integrating larger circular areas in central vision. Averaging the flow over five degrees leads to an even better fit of the model to experimental thresholds. We also found that the minimal optical flow speed direction corresponds to a maximal sensitivity of the visual system, as predicted by our model. The spontaneous gazing strategies observed during driving might thus correspond to an optimal selection of relevant information in the optical flow field.

## Introduction

How do humans perceive and control their motion in the environment? One performs this task on a daily basis, when walking in the street or driving on a winding road. Among other sources of information, vision plays a leading role in the control of self-motion [1], [2]. The specific curve-driving situation has been the subject of several studies which tried to identify the crucial visual cues for curvilinear self-motion [3], [4] or discuss the role of the optical flow field [5].

While this topic remains a debate, Land and Lee (1994) [6] provided a significant contribution in a driving task. They were among the first to record gaze behavior during curve driving on a road clearly delineated by edge-lines. They reported frequent gaze fixations toward the inner edge-line of the road, near a point they called the tangent point (TP). This point is the geometrical intersection between the inner edge of the road and the tangent to it, passing through the subject's position. This behavior was subsequently confirmed by several other studies [7]–[9] with more precise gaze recording systems.

**Figure 1:**
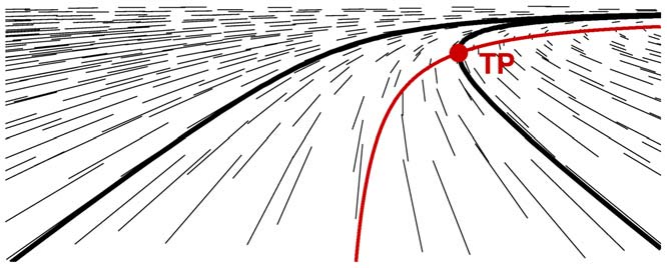
An optical velocity field generated by a circular trajectory parallel to the ground plane and aligned with road geometry. The edge-lines of the road are represented by continuous black lines and the tangent point by a red dot. The virtual line (in red) corresponds to an inversion of the horizontal component of optic flow velocity. The tangent point is the intersection between the red line and the edge-line.

All of these studies suggest that the tangent point area contains useful information for vehicular control. Indeed, the TP features specific properties in the visual scene. First, in geometrical terms, the TP is a singular and salient point from the subject's point of view, where the inside edge-line optically changes direction (Figure 1). Secondly, the location of the TP in the dynamic visual scene constantly moves, because its angular position in the visual field depends on both the geometry of the road and the car's trajectory. Thus, this point is a source of information at the interface between the observer and the environment: an ‘external anchor point’, depending on the subject's self-motion with respect to the road geometry. Lee (1978) [10] coined this as ‘ex-proprioceptive’ information, meaning that it comes from the external world and provides the subject with cues about his/her own movement. These characteristics (saliency and ex-proprioceptive status) indicate that the TP is a good candidate for the control of self-motion. Furthermore, the angle between the tangent point and the car's instantaneous heading is proportional to the steering angle: this can be used for curve negotiation [6], [11]. Moreover, steering control can also integrate other information, such as a point in a region located near the edge-line [12].

However, this depiction of the tangent point status is incomplete. It neglects its specificity in the optical flow array and the optical flow itself. Indeed, the observer's movement through the environment corresponds to a complex, continuous transformation of retinal images, the optical flow, which depends both on the characteristics of the observer trajectory and the three-dimensional structure of the environment [13], [14]. Although the use of optical flow for controlling self-motion flow remains a matter of debate [15], [16], we know that humans can perceive their direction of heading from optical flow patterns during rectilinear [17] and curvilinear [4] trajectories with great accuracy (typically about 1 degree). For rectilinear trajectories, Gibson [18] identified an ‘invariant’ in the optical flow array, the focus of expansion. It corresponds to a null speed in the optical flow array and specifies the movement direction. Experimental studies have confirmed that heading can be perceived from the pattern of global optical flow, and/or the location of the focus of expansion [18], [19]. Moreover, heading discrimination thresholds increase with gaze eccentricity from the focus of expansion [20], [21], which suggests that gazing in the direction where flow speed is minimal enhances the discrimination of one's trajectory.

However, the focus of expansion is not available for curvilinear trajectories. The only point where the optical velocity is equal to zero is the geometrical center of the curve [22] which is usually outside the visual field. Instead, the TP corresponds to a salient minimum of optic flow speed in the driver's visual scene when the vehicle trajectory is aligned with the borders of the road (Figure 1). In the optical flow field, the TP is the intersection between the inside line of the road and a virtual circle passing through the subject position and the center of the curve. This virtual ‘reversal’ line corresponds to an inversion of the horizontal component of optic flow velocity and therefore to a minimal optical speed at a given angular elevation in the driver's visual field [22], [23] (see Figure 1 and Appendix S1).

**Figure 2:**
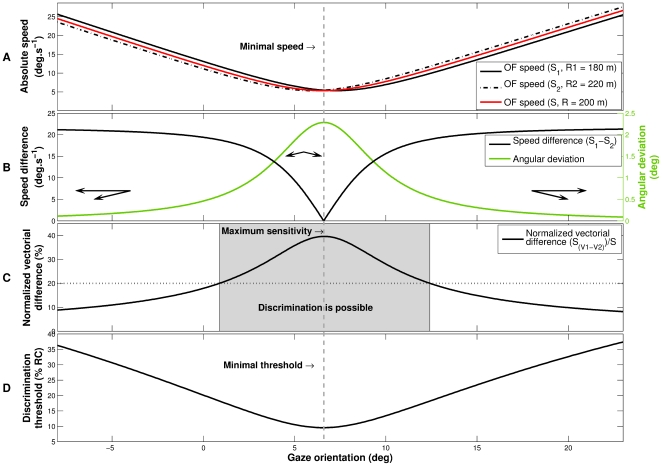
Effect of gaze orientation on local properties of optical flow. **A.** Local optical flow speed elicited by three different trajectories as a function of the horizontal gaze orientation. The optical speed for the median trajectory (S with a radius of curvature of 200 m, solid red line) reaches a minimal speed indicated by a vertical dashed gray line. **B.** Optical flow speed difference and angular deviation between the flow vectors for trajectories R1 (180 m) and R2 (220 m), as function of the gaze orientation. The speed difference (black line) reaches a zero value for a minimal optical speed. For the same gaze orientation, the angular deviation (green line) between the flows vectors is maximal. Three examples of flow vectors for R1 and R2 trajectories are drawn in black. **C.** Normalized vectorial difference between local optical flow vectors as a function of the gaze orientation. The normalized vectorial difference corresponds to the norm of the vectorial difference (between the flow vectors associated to the trajectories R1 and R2), divided by the average speed. The normalized vectorial difference reaches a maximum for the same orientation of gaze as the optical flow speed minimum. A maximum normalized vectorial difference would correspond to the maximum sensitivity regarding a change in the curvature. If an observer detects a normalized vectorial difference of 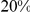 (dotted horizontal line), the gaze direction range in which the discrimination between R1 and R2 is possible will correspond to the shaded area. **D.** Discrimination threshold predictions (in percentage of difference of radius of curvature) as a function of the gaze orientation for an observer who detects a 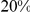 normalized vectorial difference. The gaze direction corresponding to the minimal threshold is identical to the minimal optical flow speed direction.

This description supports a new hypothesis about the status and usability of the tangent point. Its minimal optical flow speed can explain its attractiveness for the visual system. We claim here that because of its minimum flow speed, the TP is indeed an optimal location in the visual field to perceive a change in the trajectory (Figure 2).

When an observer moves along a curve of constant radius, the foveal optical flow speed is a function of his/her horizontal gaze direction (Figure 2.A.), and reaches a minimum when crossing the ‘reversal’ flow line. If the trajectory changes to a new constant radius, the Weber fraction of the foveal – local – speeds (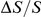; [24], [25]) will be maximal for a gaze directed toward the minimal optical flow speed (Figure 2.C.). In other words, the minimal flow speed direction would correspond to the maximal sensitivity of the visual system, regarding a change in the path curvature. If one considers this normalized vectorial difference as a cue for judging a difference of the radius of curvature, we can predict the heading discrimination thresholds for an observer (Figure 2.D.), as a function of the gaze direction in the optical flow. This model predicts that the minimal discrimination threshold is obtained for a minimal flow speed, and that the discrimination thresholds will increase with gaze eccentricity. This model only relies on the local flow structure to predict the perception of a change in the trajectory path.

Our main hypothesis is that gazing in a specific direction, which coincides with a minimal flow speed during constant curvilinear motion, will increase the observer's ability to detect a change in his/her movement direction. In that case, heading discrimination thresholds will depend on the gaze direction and therefore on the foveal optical flow speed. Within this framework, we set up an experimental study, aimed at evaluating the ability of human observers to discriminate changes in their direction of travel from optical flow patterns, as a function of their direction of gaze in a virtual environment. We simulated curvilinear self-motion parallel to a ground plane. Using random-dot optic flow stimuli of brief duration and a two-alternative forced-choice adaptive procedure, we evaluated curvature discrimination thresholds, as a function of gaze direction.

## Methods

### Ethics statement

Informed written consent was obtained from all participants prior to taking part in the experiment. Since the study involved exclusively non-invasive perceptual measurements, the study was approved by the Institute of Movement Science Laboratory Review Board. The experiment was conducted in accordance with the Declaration of Helsinki.

### Observers

Twelve young healthy observers (seven men) participated in the experiment. Their age varied from 22 to 31 years (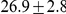 on average). Only participants with normal or corrected to normal vision were allowed to participate. Only one participant was aware of the experimental hypotheses, and none of them were ‘psychophysically’ experienced.

### Apparatus and stimuli

Stimuli were generated using an ASUS^©^ Laptop (N50VC) with in-house software using Python and OpenGL libraries in a Linux environment. The presentation of stimuli, randomization of the order of the different stimulus conditions and recording of responses (via a remote device) were all controlled by the same computer. Stimuli were rear-projected onto a large (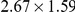 m) translucent screen. The image was projected on the screen using an LCD projector with a resolution of 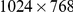 pixels, at 60 Hz. The stimuli were displayed on the lower half of the screen delimited by the horizon. We oriented the projector such that the maximum definition was used in the region of display (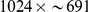 pixels). The projected image subtended 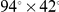 (horizontal
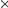
vertical) and was viewed binocularly from the unique distance of 1.25 meters. The observers' heads were stabilized by a forehead rest, in a vertical position, and centered horizontally. We adjusted the horizon height to eye level for each participant (1.33 m on average). Optic-flow stimuli simulated a curvilinear trajectory over a random-dot ground plane at constant height (parallel to the ground plane). The ground plane coincided with the floor of the experimental room.

The dots were randomly positioned for each display in a rectangular surface in the three-dimensional virtual world (i.e. on the stimulated ground plane) in order to fill the entire projected area (i.e. the screen below the horizon) during the overall simulation. Dots were single white 5.4 min of arc-wide pixels on a black background, did not expand during the simulated self-motion, and did not have a limited lifetime. The dots that left the screen were not systematically replaced, depending on the previous randomization. As such, the number of dots varied by 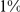 during the simulation (4100 dots were present on the screen, on average). A single display, consisting of 30 images in a lapse of 500 ms, was computed beforehand in order to insure perfect regularity of the visual stimulation frame rate.

### Psychophysical procedure

**Figure 3:**
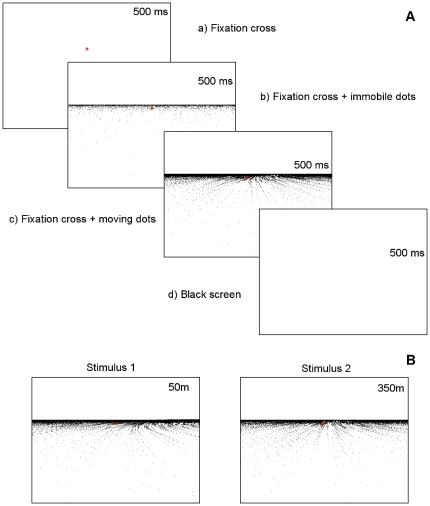
Two alternative forced choice protocol (2AFC). **A.** Schematic temporal arrangement of half of one trial. Subjects were first required to fixate a red cross on a black background. After 500 ms, random dots appeared, remained static for 500 ms and then move for 500 ms. A black screen then appeared for 500 ms, followed by the second stimulus. **B.** Comparison stimuli for a single 2AFC trial. In this example, the first stimulus displays a 50 m radius of curvature trajectory and the second a 350 m one. Observers' task was to judge which one was curved the most. Please note that colors are inverted for printing purposes.

The present experiment implemented a two-alternative forced choice (2AFC) paradigm, in order to define, for each participant and each experimental condition, the minimal difference of radius of curvature necessary to make an accurate discrimination judgment (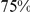 correct detection threshold) between two successively presented trajectories (Figure 3).

**Figure 4:**
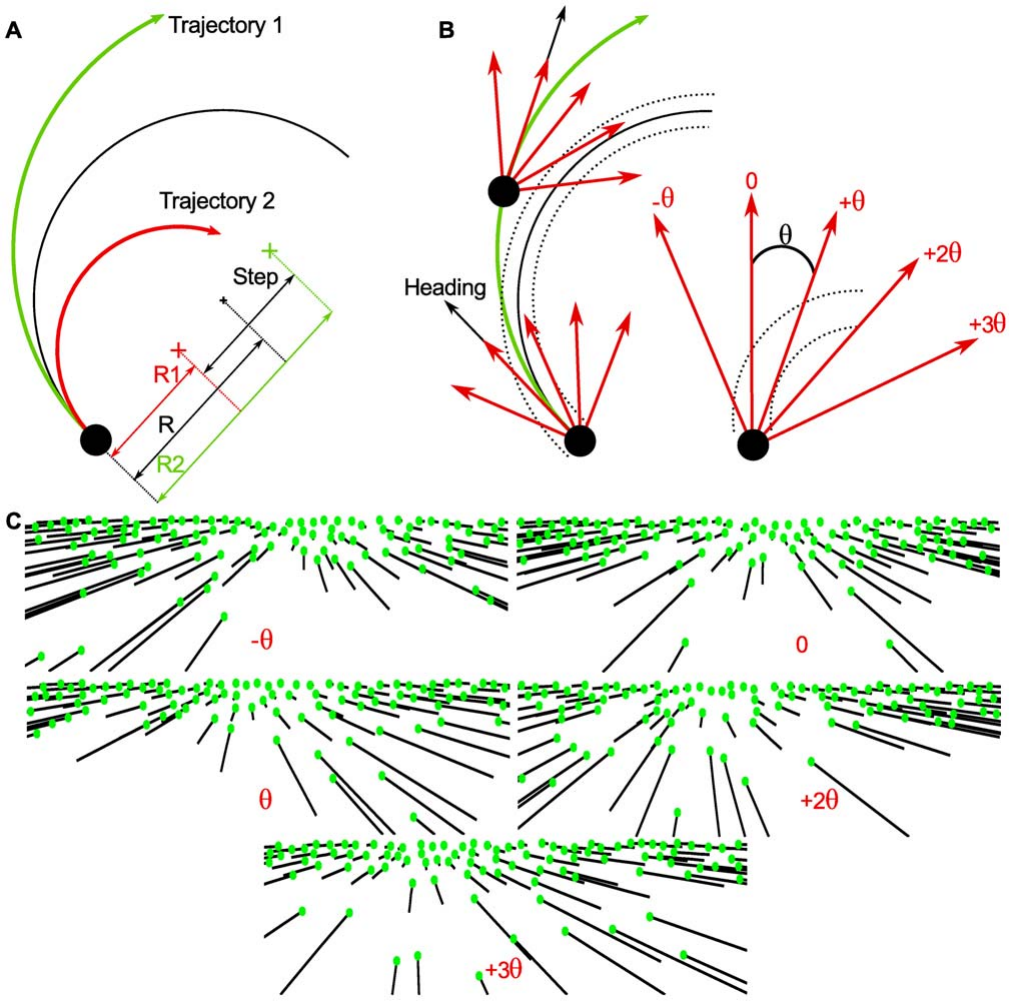
Examples of trajectories and camera orientations used in the experiment. **A.** The observers' task was to judge the relative curvature between two simulated trajectories of constant radii R1 (green trajectory) and R2 (red trajectory). These radii were centered around a target trajectory, fixed to 200 m of radius, and separated by an adjustable step quantity (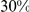 difference at the beginning, i.e. 70.58 m giving successive trajectories of 235.29 m and 164.70 m radii), following a PEST procedure. **B.** In different, experimental conditions, the camera could be rotated at five constant directions defined by multiples of the eccentricity of the tangent point direction 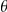. This quantity was computed from the tangent point location for a curve of 200 m radius of curvature and an ‘imaginary’ 3.5 m wide road. **C.** Representation of the flow fields corresponding to the five camera orientations for a single trajectory of 200 m of radius of curvature. A counter rotation, function of the simulated camera rotation, was applied to the virtual environment, such that the observers' actual gaze was always positioned in the center of the display screen.

Each 2AFC trial consisted of two temporal intervals; in the first interval, the dots movement simulated a curvilinear trajectory with a radius of curvature 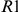; in the other interval the simulated trajectory had a different radius 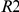, which was larger or smaller (Figure 4.A.). The order of presentation of the stimuli was randomized, so the larger radius was presented with equal probability in the two intervals. All simulated trajectories were right bends. The observers had to decide which stimulus corresponded to the most curved trajectory, i.e. had the smaller radius of curvature. No feedback was provided. At the beginning of each trial, the participant was asked to fixate a red cross at the center of the screen and 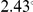 under the horizon, first displayed for 500 ms on a blank screen and visible throughout the stimulation. The fixation point was presented to stabilize the gaze. After that, the first static frame of the stimulation was displayed for 500 ms. Finally, the stimulation started and lasted for 500 ms. We chose this stimulation duration due to a double constraint; on the one hand we wanted to avoid gaze drifts from the fixation cross by minimizing display duration; and on the other hand we wanted to match the limit of temporal integration for random-dot patterns with some leeway (300 ms for rectilinear displays; [26]). An interval of 500 ms (blank screen) separated the two sequences of a trial. At the end of the second presentation, the participant was instructed to answer as fast as possible with a remote controller. As soon as the participant's answer was recorded, next trial was presented with a new set of radii parameters. If a given difference between the two radii was too easy to discriminate for an observer, the difference of radius was reduced and, inversely, was increased if too difficult.
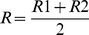
(1)When 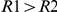, we can then express the relative difference between the two radii 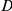 as:
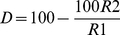
(2)


We used a PEST algorithm [27] to ‘staircase’ the relative difference between the radii of curvature that yielded 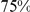 correct discrimination performance. The initial step size was fixed to 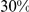. The smallest PEST step size was 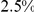. In order to achieve a stable threshold measurement, observers performed six repeated runs of 70 trials for each condition of gaze orientation (see below). We fitted individual psychometric data with a Weibull function (linear optimization in Matlab^©^) to determine the radius of curvature difference yielding 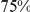 correct performance. The Weibull function has the following definition
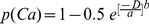
(3)with 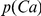 the probability of correct answer, 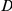 the relative difference between the two radii, 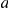 and 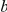 the inflexion point and the slope of the curve, respectively.

### Camera/gaze orientation

Although actual gaze direction was kept constant on the screen across all conditions (i.e. the observer was asked to fixate a target located at the center of the screen), the gaze orientation of the observers in the virtual environment was manipulated between experimental conditions and kept constant during a given condition (Figure 4.B.). We simulated multiple gaze directions in the horizontal plane by defining five constant orientations of the camera. We could have instead, displayed fixation targets at specific locations on the screen. We chose to rotate the camera – and not gaze – to avoid the potential use of extra-retinal information related to gaze direction, and to keep the same symmetrical area of stimulation on each side of the gaze position. The five conditions of orientations of the camera were 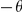, 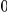, 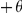, 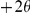, 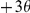, with 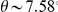. The zero direction corresponds to a simulated gaze direction aligned with instantaneous heading – straight ahead – (Figure 4.B.). The 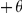 direction matches a local minimum of optical flow at the fixation cross (and gaze position) on the screen. This quantity was chosen from an ‘imaginary’ tangent point location for a curve of 200 m radius of curvature and a 3.5 m wide road. In fact, no real tangent point was present in our stimuli because no edge-lines were displayed. However the 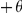 direction intersects the line of horizontal reversal of the flow (pure vertical flow) at a given horizontal position on the screen, and would also match an inside edge-line (with a lateral distance of 1.75 m to the right of the observer) at the TP location (see Appendix S1).

Gaze orientations are intimately related to different foveal flow speeds. The minimal flow speed always matches the 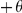 orientation, and this speed will be maximal at 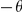 and 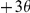 orientations. The flow fields corresponding to the different gaze orientation conditions are represented in Figure 4.C.

The main goal of orienting the gaze was to present different foveal flow speeds, with a minimum in the 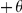 condition relative to other conditions of gaze orientation. The 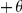 corresponded to a minimal flow speed at a given height on the screen, for a target trajectory of 200 m of radius of curvature. In the psychophysical procedure, stimuli always corresponded to a non-zero difference of radius between two successive trajectories, and trajectories of 200 m radii were never presented. When the radius of the trajectory was higher than 200 m, the horizontal position of the minimal flow speed was less eccentric, to the left of the fixation point. This effect was seizable at the beginning of the procedure, for 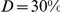 (radii of 235.29 m and 164.70 m). In this case, the position of the flow minimum was shifted horizontally by 2 degrees. However, even with these radius differences from 200 m, the experimental conditions of gaze rotation (i.e. 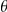 variation) were clearly differentiated.

During each experimental session, five blocks were presented in a randomized order, each one corresponding to one of a five camera orientation condition. Each block was constituted of 70 trials using a PEST procedure to derive one discrimination threshold. The experimental session was repeated six times. The entire experiment lasted 3.5 hours per observer, for a total of 2100 judgments. For analyses, we only considered the mean curvature discrimination thresholds of the last two sessions, for which a stabilized discrimination performance was reached.

### Optical flow-based model of discrimination – rationale

The objective of this model was to predict the discrimination thresholds between two trajectories, with radii of curvature 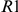 and 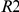. The main assumption of this model was the following: a trajectory discrimination task amounts to the discrimination of angular speeds [24], and discrimination performance is a function of the relative angular speed. This discrimination is considered to be well modeled by a Weber fraction law [28]:
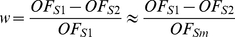
(4)with 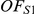 and 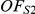 the local/foveal optical flow angular speed at the gaze position for R1 and R2 radii, respectively; 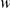 a constant (between 0 and 1, depending on the participant's discrimination performance); and 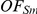 the average speed of 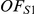 and 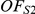.

By setting 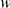 to 0.2 (i.e. 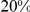 of relative flow speed) and from equations (1) and (2), we can express the curvature discrimination threshold 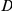 as a function of gaze orientation (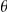), the angular speed (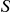) and the target radius of curvature (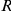, see equation (1)).
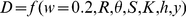
(5)where 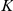 corresponds to the focal length, 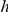 denotes the eye height from the ground plane and 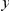 represents the vertical position of the fixation point on the screen. The complete formulation of the model can be found in the Appendix S1. The numerical resolution of the model (equation (5)) predicts that the discrimination thresholds will be lower when the observers' gaze orientation is directed toward a region of minimal optical flow speed (Figure 2).

## Results

### Discrimination thresholds

**Figure 5:**
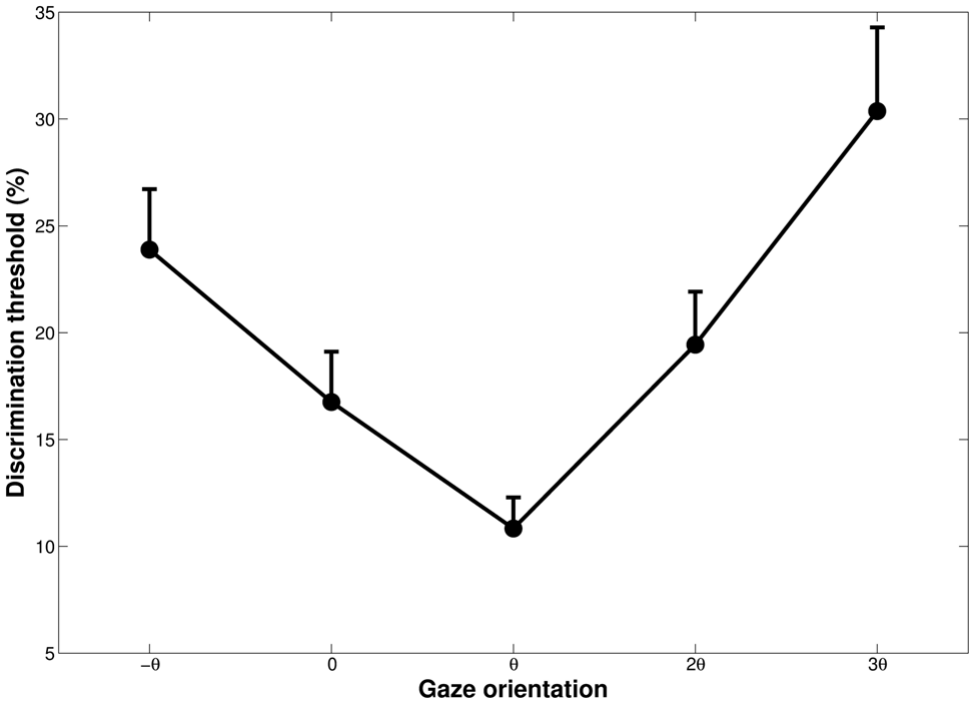
Mean percentages of path curvature discrimination thresholds, as a function of gaze orientation. The threshold is the percentage of difference between radii of curvature that observers were able to discriminate with 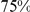 of correct responses. The 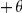 direction corresponds to the minimal optical flow velocity and to the best discrimination performance. Bars indicate between-subjects standard error.

The curvature discrimination thresholds (expressed as the percentage of difference between radii of curvature that observers were able to discriminate at a 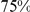 correct threshold) between the simulated trajectories in each condition are shown in Figure 5.

The mean thresholds were measured to be 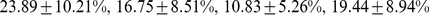 and 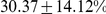 for simulated gaze rotations of 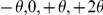 and 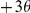; respectively. A one-way repeated analysis of variance revealed a large effect of gaze direction [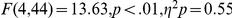]. The partial Eta squared indicated that gaze direction itself accounted for 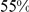 of the observed variance. Newman-Keuls post-hoc tests showed no difference between 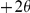 and 0 directions and between 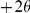 and 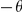 directions; but significant differences between all other orientations. The condition in which the gaze was oriented toward 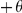 corresponded to the minimal discrimination thresholds and the 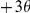 direction to the maximal thresholds. An analysis of individual results showed that the best discrimination performance was observed for the minimum of optical flow speed direction (i.e. the 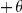 gaze orientation) for 11 participants out of 12, the remaining one had the best discrimination for 0 direction (i.e. aligned with instantaneous heading). We also noted large between-subjects variations of thresholds, with a doubled mean threshold level from the best observer to the worst.

### Local optical flows characteristics at experimental thresholds levels

**Figure 6:**
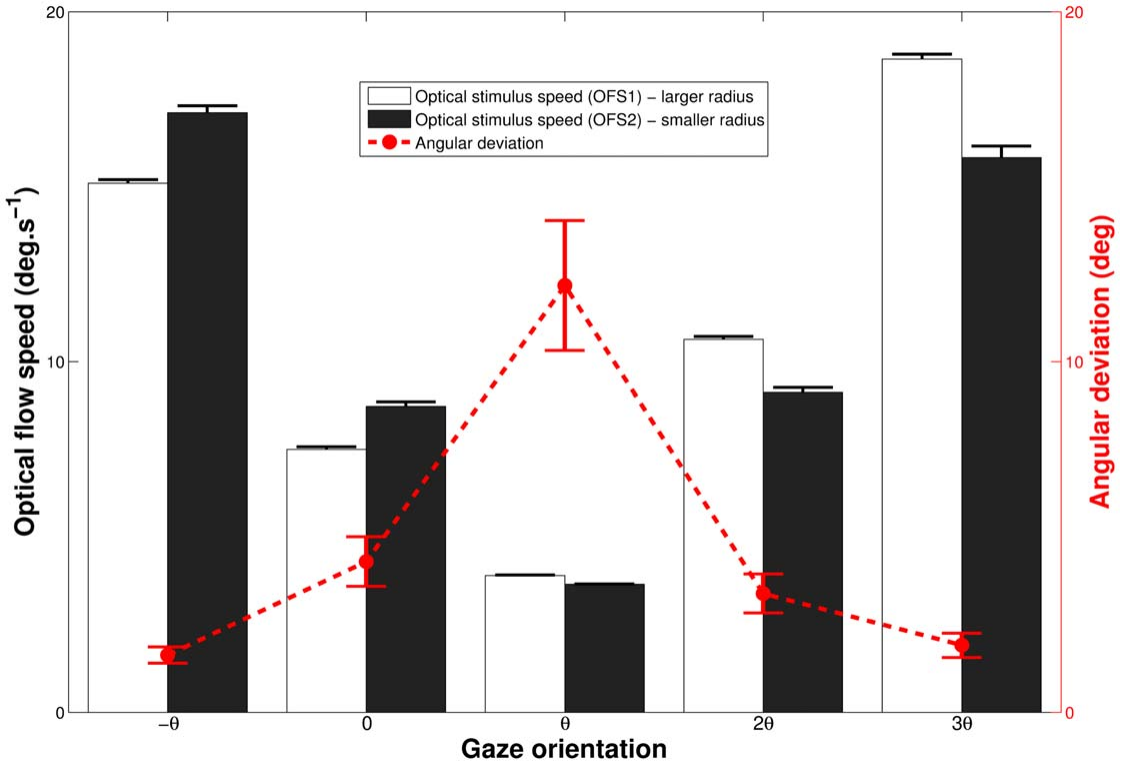
Mean experimental characteristics of optical flows as a function of gaze orientation. The optical flow speeds at the fixation position for the two stimuli are presented here; for the largest radius of curvature trajectories (white histogram); and for the sharpest one (black histogram). The absolute angular deviation between the two optical flow vectors is also represented (dashed line). Bars indicate between-subjects standard error.

For each participant and in each gaze rotation condition, we computed optical flow vectors at the cross position on the screen from equation (2) and optical flow equations (see Appendix S1). The main results can be seen in Figure 6.

The speeds of the two optical flows were minimal for the 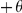 gaze orientation. An ANOVA analysis revealed a large effect of gaze direction on the optical flow speed in the largest gaze orientation stimuli [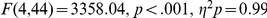]. Newman-Keuls indicated that all speeds were different from one other, with higher speed for 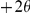, as compared to 0 rotations, and for 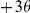, as compared to 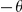 rotation. The optical flow speeds for smaller curvatures showed a quasi symmetrical result, with a large main effect [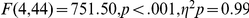], and higher speed for 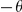 than for a 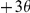 rotation. We observed only one non-significant difference between 0 and 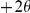 rotation condition. It is not surprising that the average speed of the two optical flow speeds was strongly affected by gaze rotation [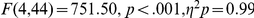] with differences revealed by Newman-Keuls between each rotation. The speed was on average, of 16.08, 8.19, 3.77, 9.83 and 17,17 degrees per second for respectively 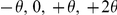 and 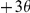 gaze rotations. The angular deviation between the two optical flows vectors was also computed. The ANOVA showed a large effect of Gaze direction [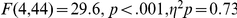] but post-hoc tests indicated that this deviation was only significantly higher for the 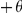 orientation.

### A model of trajectory discrimination based on local optical flow analysis

**Figure 7:**
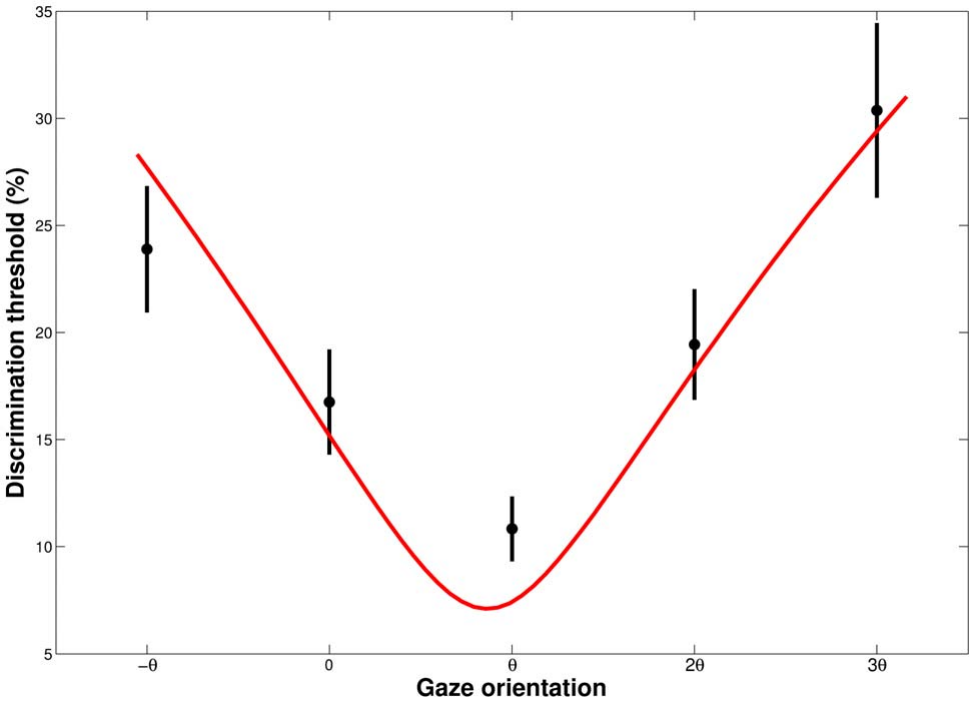
Predicted thresholds from the model (red line) and averaged discrimination thresholds (dots). The curvature discrimination thresholds between the visual trajectories are represented as a function of gaze orientation. Average data from all subjects are shown in black with the bars indicating between-subjects standard error.

The results of the simulation model are presented in this section. For the model, the discrimination thresholds between two complex stimuli (and two radii) corresponds to a comparison between two local optical vectors, through their normalized vectorial difference. Mean results of the model are presented in Figure 7.

The model was first confronted with average discrimination thresholds. We chose a 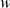 value (i.e. the normalized vectorial difference that observers were able to discriminate, equation (4)) which minimizes the root mean square error between the model and the average data. The best 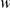 parameter found was 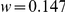, which means that, on average, the relative difference between two optical flow speeds is perceived if greater or equal to 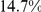. At first approximation, the simulation fits the data well, with a minimum threshold at 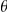 and an asymmetry around this direction, with higher thresholds for 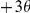 than for 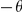 direction. A quantitative analysis revealed a good fit between the data and the model with a coefficient of determination of 0.94. We then adjusted the model to the experimental threshold of each observer. The model gives good quantitative estimates for 10 observers out of 12 (i.e. 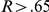).

The model thresholds were compared to the experimental ones through an individual Pearson Product-Moment Correlation Coefficient (see Table 1). The correlation of the model and the data was spread from 0.45 to 0.96 with an average of 0.77. The model only failed to explain the results for two of the participants in one gaze direction.

**Table 1:** Individual comparison between the model and the experimental thresholds.

Subject	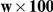	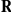	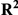
1	18.5	0.83	0.68
2	21.5	0.85	0.72
3	22	0.52	0.27
4	9.6	0.65	0.42
5	8.7	0.84	0.70
6	19.9	0.96	0.93
7	6.9	0.94	0.88
8	8.4	0.45	0.20
9	22	0.91	0.82
10	13.5	0.80	0.65
11	21.6	0.76	0.58
12	6.9	0.76	0.58
**Mean**	**14.9**	**0.77**	**0.62**
**Fit. Mean**	**14.7**	**0.97**	**0.94**

The model was fitted to the data (for the five values of gaze orientation), for each subject by seeking the best 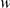 that minimized the root mean square error of the model over the data. The best 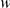 values, the Pearson R and its square values are presented for each observer. The last line corresponds to a model obtained from average threshold values of the population.

### Discrimination modeling with large flow integration areas

The model presented in the previous section relies on the computation of optical flow at a single point on the screen. However, a punctual optical flow cannot correspond to a physiologically plausible area to consider for motion processing. Moreover, the optic flow cannot be reduced to the local optic flow. We therefore evaluated the thresholds predicted by the model by computing the optical flow on larger circular areas: local, 1, 2, 3, 4, 5, 6, 7, 8, 9, 10 and 20 degrees centered on gaze position on the screen. The model was confronted with average discrimination thresholds. A slightly different method was used to obtain each prediction (see Appendix S1). However, we again chose a 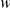 value which minimizes the root mean square error between the model (for each integration size) and the average data. The model thresholds were compared to the experimental ones through an individual Pearson Product-Moment Correlation Coefficient and root mean square error (RMSE, see Table 2).

**Table 2:** Comparison between different areas of optical flow integration in the model.

Area size (deg)	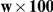	RMSE	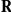	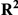
0 (local)	14.75	2.48	0.969	0.938
1	14.75	2.47	0.967	0.936
2	14.5	2.22	0.969	0.939
3	13.75	1.82	0.974	0.948
4	12.5	1.33	0.983	0.966
**5**	**10.75**	**1.20**	**0.984**	**0.968**
6	10	1.30	0.981	0.961
7	8.5	1.77	0.966	0.933
8	7.25	2.17	0.951	0.904
9	6.5	2.52	0.935	0.874
10	5.75	2.89	0.915	0.837
20	2.5	4.42	0.864	0.746

The model was fitted to the data (for the five values of gaze orientation) for the averaged experimental thresholds by seeking the best 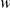 that minimized the root mean square error (RMSE) of the model over the data. Different sizes of optical flow integration areas were tested, from a single foveal point to a disc with a diameter of 20 degrees, centered on the gaze position. The best 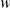 values, the RMSE of the model over the data, the Pearson R and its square are presented for each integration size. The bold line indicates the best integration area (smallest RMSE), achieved for a 5 degree area.

**Figure 8:**
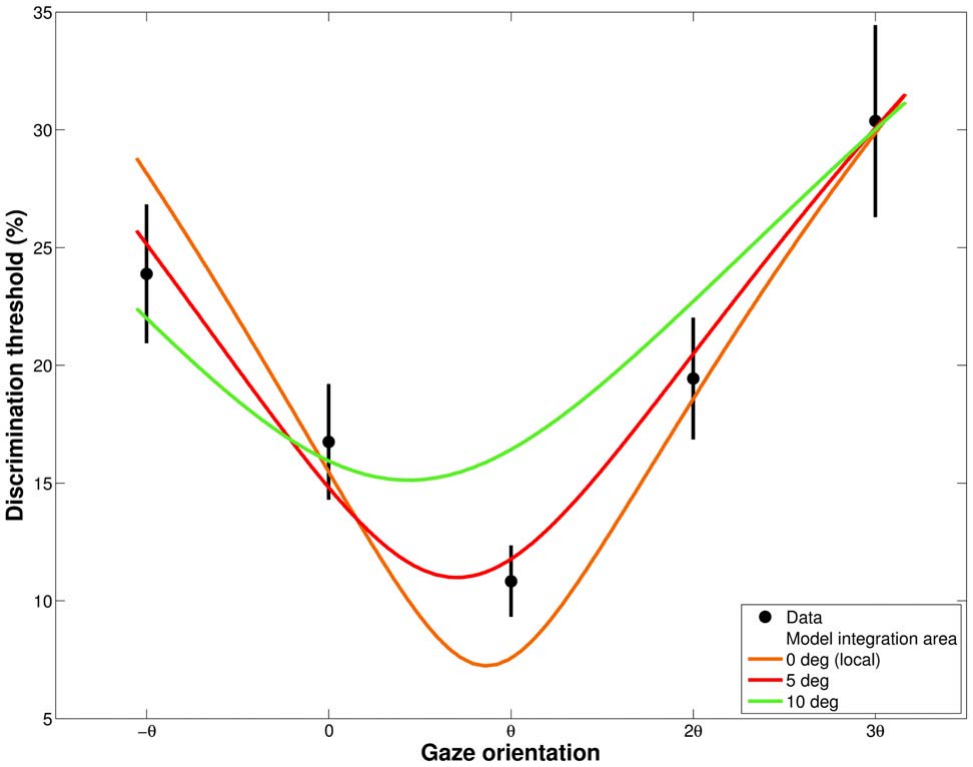
Comparison between model predictions for different areas of optical flow integration. The path curvature discrimination thresholds are represented as a function of gaze orientation. Average data from all subjects are shown in black with the bars indicating between-subjects standard error. The model predictions are represented by colored solid lines, from 0 degrees of integration (i.e. punctual optical flow) to 5 and 10 degree circular areas.

The agreement between experimental and predicted thresholds was equivalent for foveal and one-degree area, monotonically increased up to a five-degrees area and decreased for larger areas of flow integration. The predicted normalized vectorial difference that observers are able to discriminate (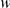 value) decreases monotonically as a function of the size of the integration area. This result reflects the fact that averaging optical flow over larger areas increases the flow speed, and the model predicts that the higher the flow speed is, the more one is able to perceive a small normalized vectorial difference. The best integration for this model is achieved for an area of five degrees. A comparison between predicted thresholds with local, five degree and ten degree area of integration is presented on Figure 8. Increasing the size of the integration area distorts the curve of predicted thresholds, leading to an asymmetry and higher thresholds for 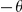 than for a 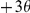 rotation.

## Discussion

The present experimental study aimed at evaluating the ability of human observers to discriminate self-motion curvature paths from optical flow patterns, as a function of their gaze direction in a virtual environment. We simulated curvilinear self-motion across a ground plane. Using random-dot optic flow stimuli of brief duration and a two-alternative forced-choice adaptive procedure, we evaluated path curvature discrimination thresholds, as a function of gaze direction. We hypothesized that discrimination threshold would be a function of the gaze orientation and therefore of the optical flow speed, and would reach a minimum for a gaze oriented toward a region of minimum flow speed.

### Discriminating is easier in region of minimal flow speed

The observed pattern of experimental thresholds suggests the importance of gaze direction for judging curvilinear heading. As predicted, the optical flow speed was a function of gaze eccentricity. The flow speeds associated to the observed experimental thresholds were minimal for a gaze orientation of 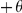 and increased with simulated gaze eccentricity. The minimum speed at 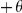 coincides with a minimum of the speed difference between the two stimuli (Figure 6). One might think that a minimal speed difference should lead to an increase of the discrimination thresholds. In this case, although the speed is observed to be minimal at 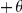 direction, a small speed difference should be indiscernible by the observers. However, the angular deviation between the flow vectors is maximal for the 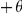 direction, and this compensates the effect of the small speed difference on the discrimination thresholds. As a consequence, we believe that the normalized vectorial difference between flow vectors is used by observers to detect differences in the characteristics of two optical flow patterns. This is corroborated by the fact that the discrimination thresholds were minimal for the gaze orientation 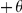, and have the same dependence on 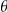 as the optical flow speed.

Our results are fully compatible with other experimental studies evaluating heading perception during rectilinear motion. The first of these was conducted by Warren & Kurtz [20], who manipulated the eccentricity of the focus of expansion from a fixation point at the center of the screen. They observed a reduction of heading discrimination performance as the eccentricity of the focus of expansion increased. Warren & Kurtz concluded that peripheral vision does not accurately extract radial flow patterns. Crowell & Banks [21] independently manipulated retinal eccentricity (the angle between the fovea and the center of the stimulus) and heading eccentricity (the angle between the heading and the center of the stimulus). They reported a large decrease of heading judgment performance with large heading eccentricities and a smaller effect of retinal eccentricity on judgments accuracy. They concluded that the visual system is equally efficient at processing radial and lamellar flow fields. Our results extend those of previous studies to curvilinear trajectories and put forward a new hypothesis explaining the better performance observed for small heading eccentricities (i.e. when gaze is located near the focus of expansion [20], [21]) and also near the minimal optical flow speed direction in the present experiment. Altogether, these results show the influence of the foveal optical flow speed on heading discrimination.

### A Weber fraction of the foveal velocities predicts the experimental thresholds of curvature discrimination

Our model based on a Weber fraction of the foveal velocities (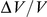), predicts the relationships between experimental thresholds and local flow speed quite well (Figure 7). This model relies on the optical flow structure, in order to predict the perception of a trajectory change. In such a model, the curvature discrimination task can be reduced to a velocity discrimination task. Both experimental data and the model show that optimal discrimination is achieved at the minimal flow speed direction (Figure 2), corresponding to the maximum sensitivity of the visual system. In a curvilinear optical flow, the flow vectors are often bi-dimensional (in the screen reference frame), and the model has to take into account both relative flow speed and direction. This implies that the model must integrate the mean speed and the norm of the vectorial difference between the flow vectors. The perception of a change in a trajectory can therefore be modeled on the basis of the local optical flow structure, and gazing toward a minimal flow speed enhances curvature discrimination. However, the optic flow cannot be reduced to the local optic flow at a single location. We therefore evaluated the model with larger circular areas for optic flow computation (Figure 8). We show that averaging the flow over five degrees leads to an even better accordance of the model and the experimental thresholds.

In the present study we do not discuss which part of the retina is involved in the perception of heading (see [20] for a review). However, we show that as far as central vision is concerned, the visual system cannot discriminate lamellar flows as precisely as radial ones. Actually, the discrimination degradation from pseudo-radial (more present in the 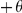 direction for example) to lamellar flow results from a single mechanism, involving optic flow speed differences. We also demonstrate that perceptive thresholds are a function of the foveal and parafoveal speed. The main local flow difference between two gaze orientations resides in the vector magnitude and not in the flow structure (lamellar or pseudo-radial). When the gaze in oriented farther away from the 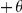 direction, the flow vectors present a lamellar structure, but above all, a higher speed. Our model shows that a single mechanism can explain the difference in performance between lamellar and radial flows.

### A new perspective for tangent point gazing strategies

Following the study by Land and Lee [6] in 1994, a large number of studies have reported a gaze fixation behavior toward the tangent point (TP; [7]–[9]) during curve driving. Various explanations can be put forward to explain this behavior. The TP is a singular and salient point from the subject's perspective and its location reflects both the road geometry and the movement direction [11]. These features indicate that the TP is a good candidate for controlling self-motion. Furthermore, the TP angle (the angle between the tangent point and the car's instantaneous heading) is proportional to the steering angle: this can be used for curve negotiation [6], [11], [12]. A simple control law to steer in curves would be to keep this angle constant, irrespective of its exact value. However, we have shown that the TP direction has other key characteristics in the optical flow field, such as being a local minimal flow speed location. The present results show that path curvature discrimination is enhanced when gaze is directed toward a region of minimal optical flow velocity. As a consequence, the spontaneous gazing strategies observed during driving might correspond to an optimal selection of relevant information in the optic flow field, and the TP could be the best location in the dynamic optical array to perceive a change in trajectory. This hypothesis is consistent with most ecological situations; the minimal optical flow and the gaze direction often matches the movement direction (for rectilinear trajectories [18]) or the future path [29], which corresponds to areas of low flow speed, such as the focus of expansion or the tangent point.

However, edge-lines clearly provide visual guidance to drivers steering around a curve [30] and these were not displayed in the present experiment. Further exploration of the interaction between optical flow structure and edge-lines is necessary to clarify the influence of the flow speed in more realistic situations. In particular, in the presence of continuous untextured edge-lines, in the case where the trajectory is perfectly aligned with the road, the inner edge-lines will ‘assume a steady state appearance’, as quoted by Gordon [23]. In that case, we can suppose that edge-line angular motion will become part of the steering control process. Moreover, we used a perceptual task with constant radii stimuli and this approach can only provide indirect support of the use of TP in steering control.

### Gaze movements and optical flow

In the current experiment, subjects were required to fixate a cross during curvilinear optical flow stimulation. If there were no fixation point to stabilize gaze, eye movements would be induced by the flow field [31]. It is well known that uni-directional optic flow triggers an optokinetic nystagmus (OKN), consisting of a succession of tracking movements in the direction of visual motion (slow phases of OKN) and fast resetting saccades in the opposite direction. Between two saccades, slow eye movements occur in order to stabilize the retinal image. OKN has already been observed in complex optic flow displays [32]. Moreover, the presence of OKN was observed in the context of simulated rectilinear self-motion of the macaque monkey [33] and humans [34], featuring radial optic flow patterns (see also [35]). In a recent study, we showed that OKN is also elicited by curvilinear optic flow during high speed curve driving [36].

If gaze direction is kept constant (as was intended in the present experiment, by using a fixation cross) the retinal flow field is equivalent to the optical flow field. On the contrary, any gaze rotation (e.g. gaze tracking an environmental element) adds a retinal slip to the optical flow structure and distorts the retinal flow field. In this case, the direction of locomotion could be determined 1) with visual information alone [19], [37] or 2) must involve extra-retinal eye movements signals [38]–[40]. Unbiased heading detection can only be performed with small speeds of simulated gaze rotation (below 6 deg/s in [41]) for simulated rectilinear self-motion. In our experiment, the 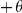 condition was the sole direction featuring a foveal flow speed under 6 deg/s (Figure 6).

Because of the presence of OKN in ‘natural’ conditions, retinal and optical flow are not identical. In this case, looking away from the zone of minimal optical flow will result in slow pursuit eye movements, reducing foveal retinal flow speed. However, it has been shown [33], [34], [36] that the slow-phase gain of OKN is never perfect (being on average equal to the two thirds of the foveal optic flow speed; *e.g.* gain of 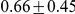 in [36]). This might result in a local minimum of foveal retinal flow speed. But since the tracking gain is below unity, it still remains that looking in a zone of minimal foveal optical flow speed will always result in a foveal retinal flow speed inferior to that resulting from looking anywhere else. In this study, we show, in gaze fixation conditions, that discrimination performance is optimal for minimal foveal flow. As such, we claim that, in more natural conditions with less constrained gaze movements, looking at the zone of minimal optical flow is the best strategy. However, the speed of the simulated self-motion is a key parameter in our model and requires further experimental investigation, in conjunction with free gaze situations.

### Conclusion

In conclusion, the visual perception of self-motion is not equally precise throughout the visual field. A given gaze direction of the moving observer corresponds to a single local flow velocity. This velocity affects curvature discrimination thresholds, which are minimal for a gaze directed toward a local minimum of optic flow speed. A model based on Weber fraction of the foveal velocities (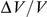) correctly predicts the relationship between experimental thresholds and local flow velocities. This model was also tested for an optic flow computation over larger circular areas and averaging the flow over five degrees leads to an even better fit of the model to the experimental thresholds. We found that a minimal speed direction corresponds to the maximal sensitivity of the visual system, as predicted by our model. Therefore, the spontaneous gazing strategies observed during driving (e.g. the tangent point fixation behavior) might correspond to an optimal selection of relevant information in the optic flow field. These findings are consistent with ecological phenomena: the minimal flow speed often matches both movement and gaze direction.

## Supporting Information

Appendix S1 **Appendix and supporting figures.** In this appendix, we first demonstrate that the TP corresponds to a local minimum of optical speed, and coincides with the intersection of the inside line of a road and a virtual circle formed by all the points where the horizontal component of the optical flow is reversed. The optical flow computation method employed in the paper is then extensively explained. In a third part, the complete formulation of the optical flow-based model of discrimination is described, as well as the fit of the model to the data. Finally, a version of the model taking in account larger optical flow integration areas is shown.
(PDF)
