# Supplementary material for: Path Curvature Discrimination: Dependence on Gaze Direction and Optical Flow Speed
Source: PLoS One. 2012 Feb 29;7(2):e31479. doi: 10.1371/journal.pone.0031479 (PMC3290598; doi:10.1371/journal.pone.0031479)
Supplement: Appendix S1 — Appendix and supporting figures. In this appendix, we first demonstrate that the TP corresponds to a local minimum of optical speed, and coincides with the intersection of the inside line of a road and a virtual circle formed by all the points where the horizontal component of the optical flow is reversed. The optical flow computation method employed in the paper is then extensively explained. In a third part, the complete formulation of the optical flow-based model of discrimination is described, as well as the fit of the model to the data. Finally, a version of the model taking in account larger optical flow integration areas is shown. (PDF) [file pone.0031479.s001.pdf]

OPEN ACCESS Freely available online PLoS one

Appendix S1

Path curvature discrimination: dependence on gaze direction and optical flow speed

Colas N. Authié[1,*], Daniel R. Mestre[1],
1, CNRS & Aix-Marseille Univ, UMR 7287 Institut des Sciences du Mouvement, Marseille, France
∗ **E-mail: colas.authie@gmail.com**

A1. Tangent point and optical flow

In the present study, we hypothesized that the tangent point (TP) is the best location in the dynamic optical array to perceive a change in the trajectory, because of its minimal optical speed. In this appendix, we demonstrate that the TP corresponds to a local minimum of optical speed and coincides with the intersection of the inside line of a road and a virtual circle corresponding to an inversion of the horizontal component of optic flow velocity.

Optical flow computation - Gordon's method

We will work with Gordon set of equations that describe the optical flow [1]. The coordinate system is shown in Figure 1.A. The eye is at the origin. Distance ahead from the eye is represented by x, to the side (horizontal distance) by y, and up and down by z. Distance from the eye to a terrain or other point is represented by $p = \sqrt{x^2 + y^2 + z^2}$, whose projection on the xy plane is $r = \sqrt{x^2 + y^2}$. θ is the horizontal

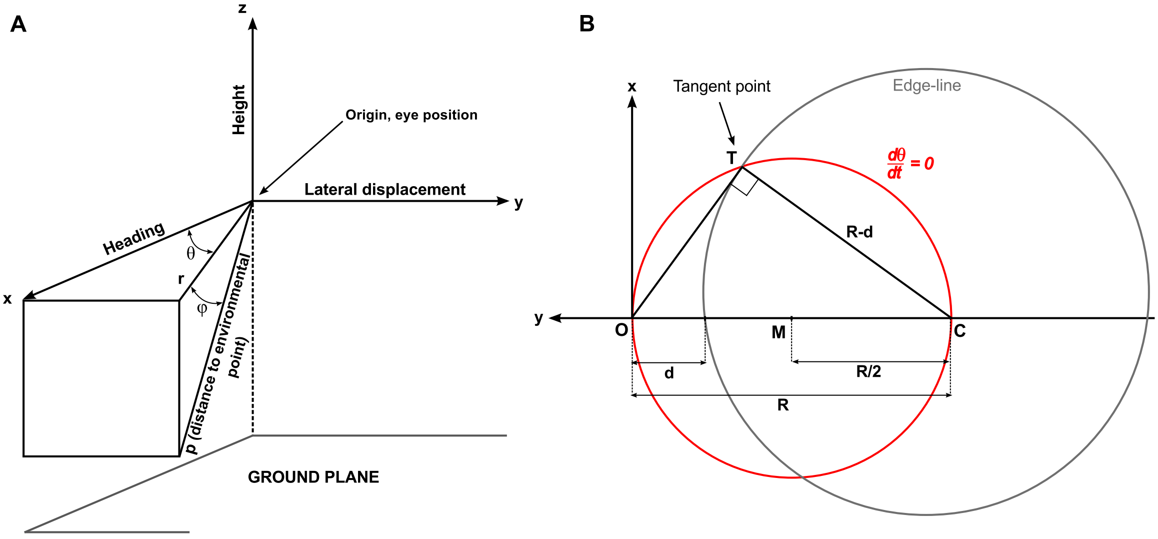

Figure 1. Horizontal reversal flow line and tangent point. A. Basic coordinate system. Adapted from Gordon [1]. **B.** Geometrical representation of the horizontal reversal line ($\frac{d\theta}{dt} = 0$) of the flow (red circle with center M and radius $R/2$) and the inner edge-line of a road (gray circle with center C and radius $R - d$). We consider a trajectory of constant radius R around the center of rotation C. The tangent point position T is the intersection between these two circles; d corresponds to the distance between the observer O position and the inner edge-line.

angle of a given environmental element and ϕ is the vertical angle in the visual field. To describe the moving environment, the position and induced movement of objects expressed in Cartesian coordinates (x, y, z, dx/dt, dy/dt, dz/dt) must be translated into spherical coordinates (θ, ϕ, $d\theta/dt$, $d\phi/dt$, $d^2\theta/dt^2$, $d^2\phi/dt^2$) of the observer's visual world.

The optical flow velocity on a horizontally curved path, with a fixed radius of curvature R, can be decomposed into a horizontal component $\frac{d\theta}{dt}$ and a vertical one $\frac{d\phi}{dt}$. The horizontal speed component is defined as:

$$\frac{d\theta}{dt} = \left(\frac{-y}{r^2} - \frac{1}{R}\right)\frac{dx}{dt} \quad (1)$$

The vertical speed has the following expression:

$$\frac{d\phi}{dt} = \left(\frac{-xz}{rp^2}\right)\frac{dx}{dt} \quad (2)$$

Horizontal reversal flow line

A simple observation of the oriented vectors of the optical flow reveals that a part of them are oriented to the left, and another part to the right, in other words that the horizontal orientation of the flow flips sign. Our first objective is to define the optical flow contour induced by this sign inversion. This corresponds to a null horizontal speed:

$$\frac{d\theta}{dt} = 0 \quad (3)$$

By replacement in equation (1), one gets:

$$\frac{-y}{x^2 + y^2} - \frac{1}{R} = 0 \quad (4)$$

Trivial algebra leads to:

$$x^2 + y^2 = -Ry \quad (5)$$

The equation (1) defines the set of points in which the horizontal speed is zero.
Now, let us consider a circle on the xy plane of radius $\frac{R}{2}$ and of center $M = (0, -R/2)$:

$$(x-0)^2 + \left(y + \frac{R}{2}\right)^2 = \left(-\frac{R}{2}\right)^2 \quad (6)$$

this equation (6) is equivalent to equation (5). The contour drawn by the horizontal reversal of optical flow is therefore a circle on the ground plane (the red circle on Figure 1.B).

Reversal flow line and tangent point

We have demonstrated that the equation of a circle defines the points with a zero horizontal speed. We will now demonstrate that this circle intersects any edge-line at the TP location. Let us consider a circular edge-line distant from the subject by a quantity d on y axis, with $0 > d > R$, for a right oriented curve (see Figure 1.B). The intersection between this circle and the reversal circle is the point T. The triangle OTC is inscribed in the reversal circle, and the segment $[OC]$ is the diameter of the circle. We can state from Thales' theorem that the angle $\widehat{OTC}$ is a right angle. The segments $[OT]$ and $[TC]$ are then orthogonal, with $[TC]$ a radius of the edge-line circle. $[OT]$ is therefore tangent to the edge-line circle. We have demonstrated that the reversal circle intersects any edge-line at the TP location.

The tangent point: a local minimum of flow speed

In this appendix, we have shown: 1) that the horizontal reversal of flow is a circle, and 2) the intersection of this circle with the edge-line circle is actually the TP. The reversal flow line – and therefore the TP – corresponds to a minimum of horizontal optical speed. However, it does not correspond to a minimum of the optical speed all over the environment: the only point where the optical velocity is equal to zero is the geometrical center of the curve [2]. In this section we will show that at a given vertical position on the screen, and for the specific field of view used in the experiment, the TP corresponds to a minimal speed in the flow. In order to do that, we computed the isoangular-speed curves under curvilinear motion (Figure 2). These speed values were obtained from a fixed sum of azimuth and elevation components, which add quadratically as follows:

$$\sqrt{\left(\frac{d\phi}{dt}\right)^2 + \left(\frac{d\theta}{dt}\right)^2} \tag{7}$$

The structure of the optical flow speed is visible on Figure 2 for a 200 meters radius trajectory. This representation shows that whatever is the edge-line position, and with the radius and field of view used in the present experiment, the TP always corresponds to a minimal optical flow speed for a given elevation.

A2. Optical flow computation

In the context of this study, we wanted to investigate the relationships between trajectory discrimination and local optical flow speed at the gaze fixation position. To achieve this, we needed to compute local optical flow motion for each gaze orientation (θ) and radius of curvature (R). We present here a new method, elaborated from previous works by Lappe et al [3] (1998) and Longuet-Higgins & Prazdny [4] (1980) which extends optical flow computation to the rotational velocities of the observer (curvilinear motion). We computed the optic flow velocity f at a foveal position (x, y) on the screen from the instantaneous translational (T_X, T_Y, T_Z) and rotational (R_X, R_Y, R_Z) velocities of the observer in the 3-D (X, Y, Z) environment (equation (8), Figure 3).

$$f(x,y) = \frac{-y}{Kh}\begin{pmatrix}-KT_X + xT_Z\\ -KT_Y + yT_Z\end{pmatrix} + \begin{pmatrix}\frac{xy}{K}R_X - KR_Y - \frac{x^2}{K}R_Y + yR_Z\\ KR_X + \frac{y^2}{K}R_X - \frac{xy}{K}R_Y - xR_Z\end{pmatrix} \tag{8}$$

where K corresponds to the focal length and h denotes the eye height from the ground plane.
In our experiment, we had only one rotation around the Y axis (Figure 3), and no translation along this axis (constant height from the ground plane). We also wanted to compute the optical flow characteristics at the horizontal center position of the screen, we can therefore set x to zero.

$$f(x,y) = \frac{-y}{Kh}\begin{pmatrix}-KT_X\\ yT_Z\end{pmatrix} + \begin{pmatrix}-KR_Y\\ 0\end{pmatrix} \tag{9}$$

The instantaneous translational speed tangent to the trajectory S (instantaneous heading direction) was kept constant in the present experiment. When changing the gaze orientation of a θ angle, T_X and T_Z are

$$T_X = S.\sin(\theta) \tag{10}$$

$$T_Z = S.\cos(\theta) \tag{11}$$

During curvilinear motion, the direction of the point of view coincides with the instantaneous heading and the rotational speed (for a trajectory with a radius R) must be

$$R_Y = \frac{-S}{R} \tag{12}$$

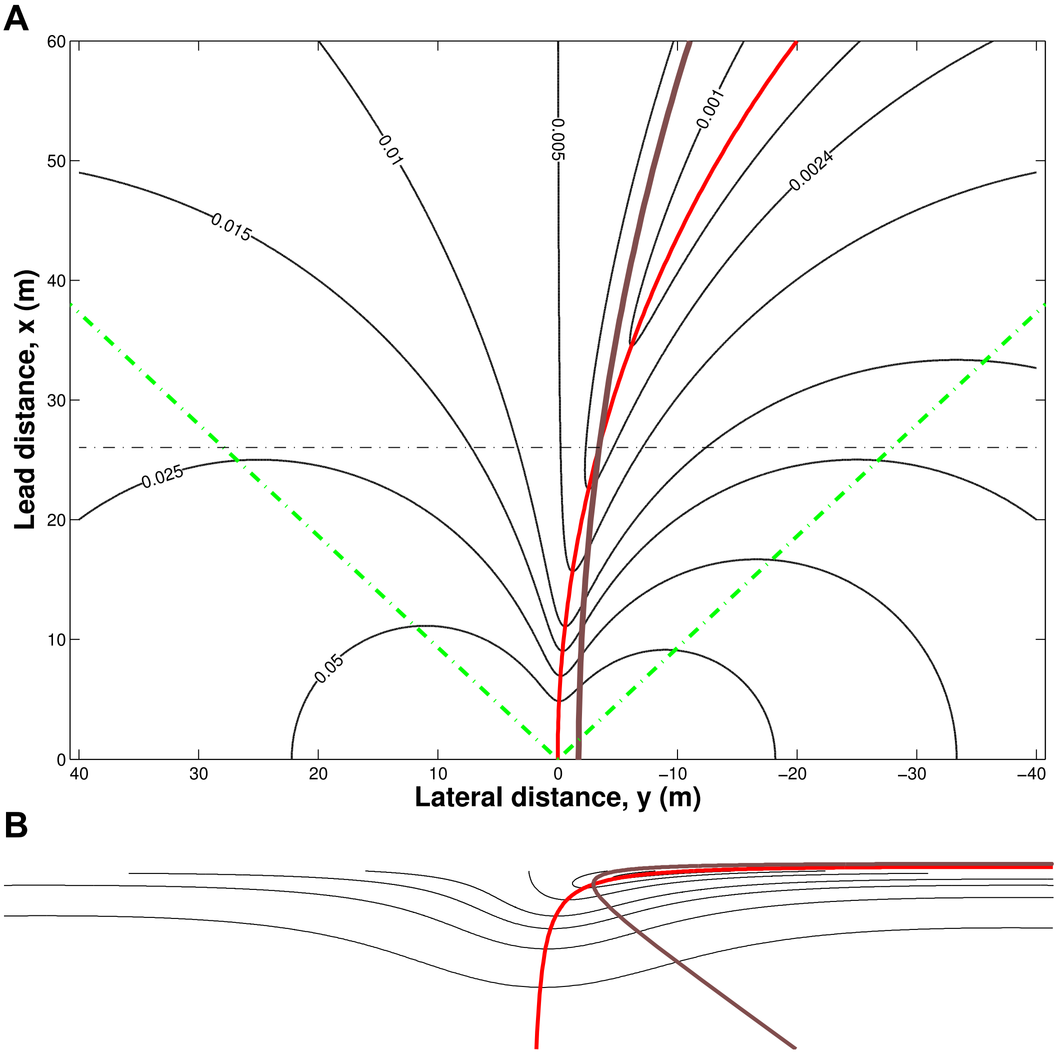

Figure 2. Isoangular-speed curves under curvilinear motion in a right curve. A. Isoangular-speed projected on the ground plane (black contours) in Gordon's coordinates (see Figure 1). The center of curvature is 200 meters to the right of origin. Angular speed in radians per second for each contour can be obtained by multiplying values shown for each curve by vehicular speed in meters per second. The horizontal flow reversal line (bold red line), an edge-line (bold brown line), the field of view (dashed green lines) and a horizontal line at the TP lead distance (dashed black line) are represented. **B.** Same isoangular-speed contours projected on the screen plane (black contours). The horizontal flow reversal line is in red and the edge-line in brown. Their intersection is the tangent point.

Substituting from equations (10), (11) and (12) into equation (9) we obtain

$$f(x,y) = \frac{-y}{Kh}\begin{pmatrix} -KS.\sin(\theta) \\ yS.\cos(\theta) \end{pmatrix} + \begin{pmatrix} K\frac{S}{R} \\ 0 \end{pmatrix} \tag{13}$$

which is the optical flow vector at the cross position on the screen depending on focal length K, eye height h, gaze orientation θ, speed S and radius of curvature of the trajectory R.

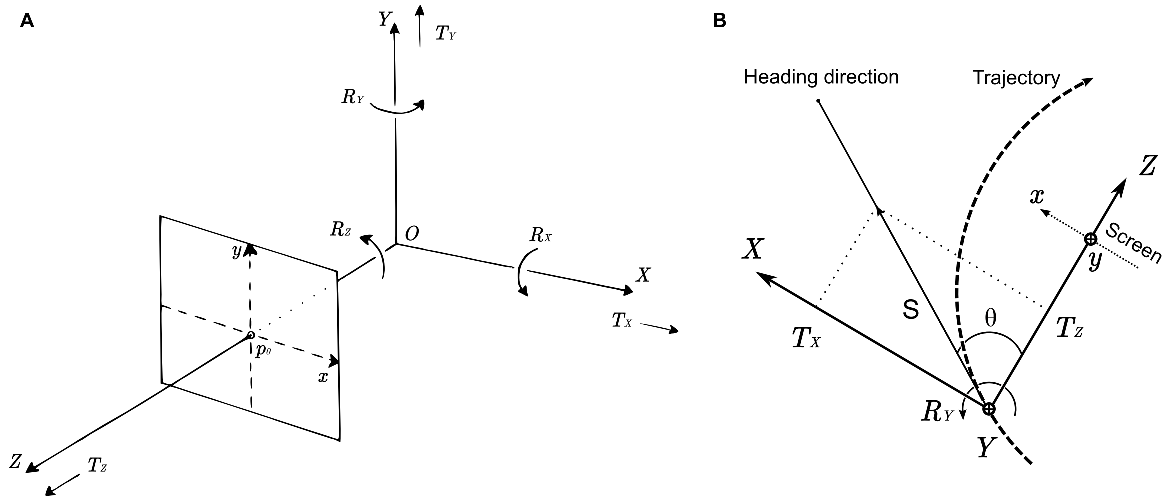

Figure 3. Coordinate system for optical flow computation. A. External coordinate system $OXYZ$ moving with the screen, and the corresponding retinal coordinates (x, y). The distance OP_0 is the focal length. **B.** The camera direction revolved around the Y axis from instantaneous heading direction. The tangent motion S was then expressed on T_X and T_Z axes.

A3. Fitting the model to the data

We know from equation (13) that an optical flow single vector is only a function of some constants (S, h, K) of gaze orientation and radius of curvature of the trajectory (θ and R, respectively). We can then write the two optical flow velocities for the two trajectories as follows

$$\overrightarrow{OF_1}\begin{pmatrix}OF_1x\\OF_1y\end{pmatrix}=\begin{pmatrix}f(\theta,R1)\\f(\theta)\end{pmatrix} \tag{14}$$

$$\overrightarrow{OF_2}\begin{pmatrix}OF_2x\\OF_2y\end{pmatrix}=\begin{pmatrix}f(\theta,R2)\\f(\theta)\end{pmatrix} \tag{15}$$

We know also from equation (1) of the paper that $R1$ and $R2$ are related by R (target radius of curvature)

$$\overrightarrow{OF_2}\begin{pmatrix}OF_2x\\OF_2y\end{pmatrix}=\begin{pmatrix}f(\theta,R,R2)\\f(\theta)\end{pmatrix} \tag{16}$$

We can now compute the optical flow speed OF_{Sm}, OF_{S1} and OF_{S2} from equations (15) and (16). The objective of the model is to keep w constant with

$$w=\frac{OF_{S1}-OF_{S2}}{OF_{Sm}} \tag{17}$$

To avoid solutions where the two optical flow vectors have the same magnitude but different directions, we choose to solve it under the form:

$$w=\frac{\parallel \overrightarrow{OF_1}-\overrightarrow{OF_2}\parallel}{\parallel \overrightarrow{OF_m}\parallel} \tag{18}$$

Combination of equations (13), (15) and (16) into equation (18) gives:

$$w=\frac{2\left|\frac{K\,S}{R2}-\frac{K\,S}{2\,R-R2}\right|}{\sqrt{\left(\frac{K\,S}{R2}+\frac{\sin(\theta)\,y\,S}{h}\right)^2+\frac{\cos(\theta)^2\,y^4\,S^2}{h^2\,K^2}}+\sqrt{\left(\frac{K\,S}{2\,R-R2}+\frac{\sin(\theta)\,y\,S}{h}\right)^2+\frac{\cos(\theta)^2\,y^4\,S^2}{h^2\,K^2}}} \tag{19}$$

We then inject this expression of w in the definition of the threshold D (equation (2) of the paper) and solve it using Taylor series. The whole resulting form cannot be printed here but is a function of the parameters:

$$D = f(w, R, \theta, S, K, h, y) \tag{20}$$

D is computed numerically for each subject in order to find the best w which minimizes the root mean square error between the model (D) and the data (experimental thresholds).

A4. Discrimination modeling with large optical flow integration areas

The model presented in the previous section relies on the computation of the optical flow at a single point on the screen. However, a punctual optical flow cannot correspond to a physiologically plausible area to consider for motion processing. Moreover, the optical flow is not reducible to the local optic flow (at gaze location). In the current section, we solve the model equation (18) given an optical flow integrated (averaged) over circular areas of variable size.
Up to now, we have been using a formulation of the optical flow at a given position ($x = 0$, y) (equation (9)); we must use a more general formulation of the flow, allowing x to vary (equation (8)). We inject the translations T_X and T_Z (equations (10) and (11)) and the rotation R_Y (equation (10)) in equation (8):

$$f(x, y) = \begin{pmatrix} -\frac{y\,(cos(\theta)\,x\,S - sin(\theta)\,K\,S)}{h\,K} + \frac{K\,S}{R} + \frac{x^2\,S}{K\,R} \\ \frac{x\,y\,S}{K\,R} - \frac{cos(\theta)\,y^2\,S}{h\,K} \end{pmatrix} \tag{21}$$

which is the optical flow vector at a given position on the screen (x, y), depending on the focal length K, the eye height h, the gaze orientation θ, the speed S and the radius of curvature of the trajectory R. We also know that the relative difference between the two radii D (i.e. discrimination threshold) is $D = 100 - \frac{100R2}{R1}$ (equation (2) of the paper). We then computed for various values of D (from 5 to 40%) and θ (from -8 to 24 degrees) the values of w from equations (18) and (21). w values were also computed with averaged values of optical flow vectors $\overrightarrow{OF_1}$ and $\overrightarrow{OF_2}$ with multiple variations of x and y on the screen, regularly spread in circular areas centered on the gaze position. The last step is to find in the two-dimensional space (D, θ), the contours of same w value, which correspond to the model predictions for a given integration area (see Figure 4). The best w contour is the one that minimizes the root mean square error by comparison of the model against the data.

References

1. Gordon DA (1966) Perceptual basis of vehicular guidance. Public Roads 34: 53–68.

2. Cutting JE (1986) Perception with an eye for motion. MIT Press / Bradford Books, 335 pp. doi: ISBN 0262031191.

3. Lappe M, Pekel M, Hoffmann KP (1998) Optokinetic eye movements elicited by radial optic flow in the macaque monkey. Journal of Neurophysiology 79: 1461–1480.

4. Longuet-Higgins HC, Prazdny K (1980) The interpretation of a moving retinal image. Proceedings of the Royal Society London B Biological Sciences 208: 385–397.

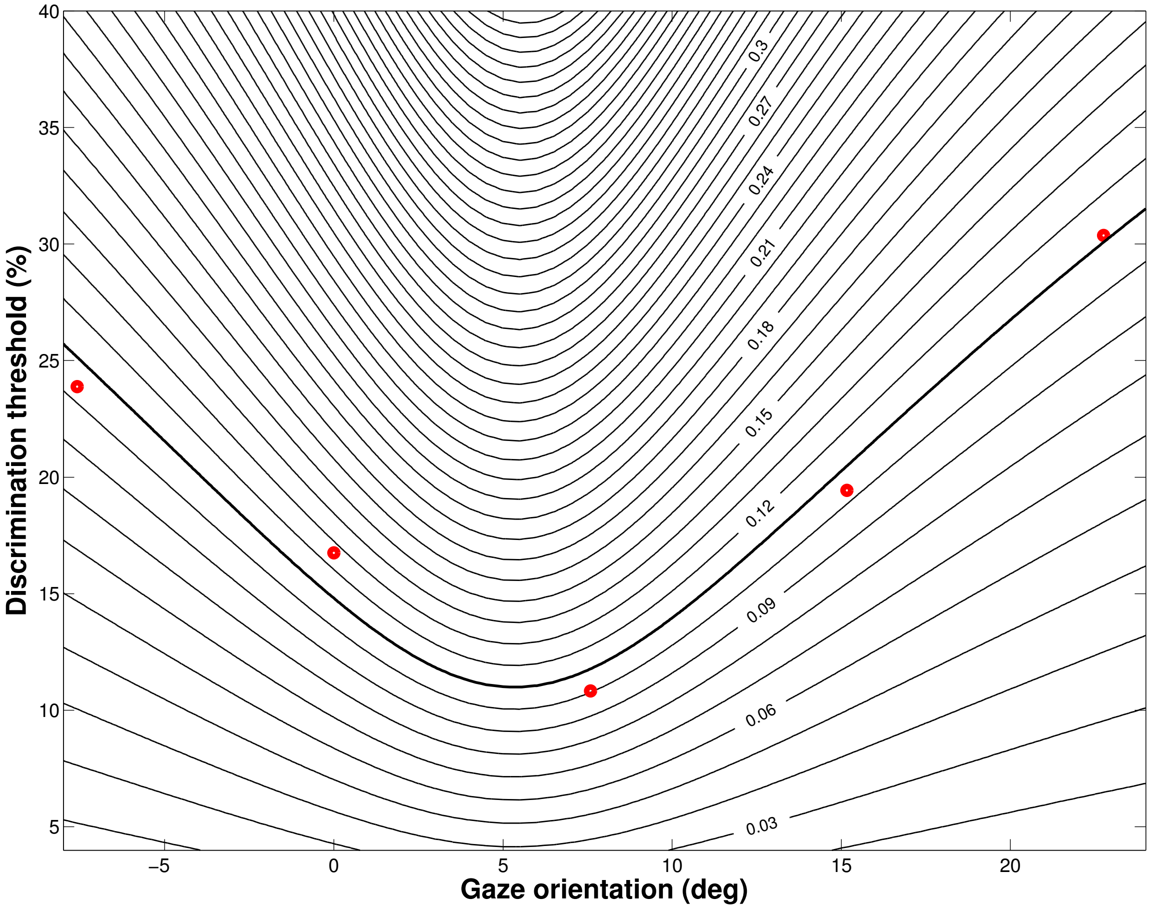

Figure 4. Predicted thresholds for the model, with an integration of the optical flow on a circular area of 5 degrees. The curvature discrimination thresholds are represented as a function of gaze orientation. Average data from all subjects are shown in red. The model predictions for different w values are represented by black solid contours. The bold black line corresponds to the w value which gives the best root mean square error between the model and the data.
